# Mitochondrial bioenergetics, metabolism, and beyond in pancreatic β-cells and diabetes

**DOI:** 10.3389/fmolb.2024.1354199

**Published:** 2024-02-09

**Authors:** Alejandra María Rivera Nieves, Brian Michael Wauford, Accalia Fu

**Affiliations:** ^1^ Diabetes Center of Excellence, University of Massachusetts Chan Medical School, Worcester, MA, United States; ^2^ Program in Molecular Medicine, University of Massachusetts Chan Medical School, Worcester, MA, United States

**Keywords:** Type 1 diabetes, Type 2 Diabetes, mitochondria, pancreatic beta cells, bioenergetics, metabolism, calcium, insulin secretion

## Abstract

In Type 1 and Type 2 diabetes, pancreatic β-cell survival and function are impaired. Additional etiologies of diabetes include dysfunction in insulin-sensing hepatic, muscle, and adipose tissues as well as immune cells. An important determinant of metabolic health across these various tissues is mitochondria function and structure. This review focuses on the role of mitochondria in diabetes pathogenesis, with a specific emphasis on pancreatic β-cells. These dynamic organelles are obligate for β-cell survival, function, replication, insulin production, and control over insulin release. Therefore, it is not surprising that mitochondria are severely defective in diabetic contexts. Mitochondrial dysfunction poses challenges to assess in cause-effect studies, prompting us to assemble and deliberate the evidence for mitochondria dysfunction as a cause or consequence of diabetes. Understanding the precise molecular mechanisms underlying mitochondrial dysfunction in diabetes and identifying therapeutic strategies to restore mitochondrial homeostasis and enhance β-cell function are active and expanding areas of research. In summary, this review examines the multidimensional role of mitochondria in diabetes, focusing on pancreatic β-cells and highlighting the significance of mitochondrial metabolism, bioenergetics, calcium, dynamics, and mitophagy in the pathophysiology of diabetes. We describe the effects of diabetes-related gluco/lipotoxic, oxidative and inflammation stress on β-cell mitochondria, as well as the role played by mitochondria on the pathologic outcomes of these stress paradigms. By examining these aspects, we provide updated insights and highlight areas where further research is required for a deeper molecular understanding of the role of mitochondria in β-cells and diabetes.

## 1 Introduction

Mitochondria are integral to pancreatic β-cell function and survival. The multifaceted role of mitochondria in β-cells encompasses pivotal processes and pathways, including mitochondrial bioenergetics and metabolism, proton leak, calcium (Ca^2+^), structural integrity, dynamics, as well as turnover/mitophagy. In this review, we delve into the nuanced roles played by β-cell mitochondria. Remarkably, an individual pancreatic β-cell is estimated to have hundreds to thousands of mitochondria due to its high reliance on mitochondrial activity. Understanding the interplay between β-cell mitochondrial structure and function is imperative in unraveling the intricate role of mitochondria in the pathogenesis of Type 1 and Type 2 diabetes (T1 and T2D). In this comprehensive review, we compare mitochondria in healthy and diabetic β-cells and highlight the evidence that mitochondria play a causal role in β-cell demise and diabetes development.

## 2 The good: mitochondrial roles in healthy β-cells

Healthy pancreatic β-cells are metabolic machines with adapted fuel preferences and metabolism required to perform their functions as glucose sensors. They contribute to systemic metabolic homeostasis by dosing the body with insulin, released via an orchestrated program of cellular excitation and metabolism. Mitochondrial activity is required for insulin secretion, β-cell expansion in response to increased functional demand, and balance of turnover and renewal/replication for maintaining β-cell mass. In the next sections, we detail the involvement of mitochondria as a homeostat in healthy β-cell function and survival.

**FIGURE 1:**
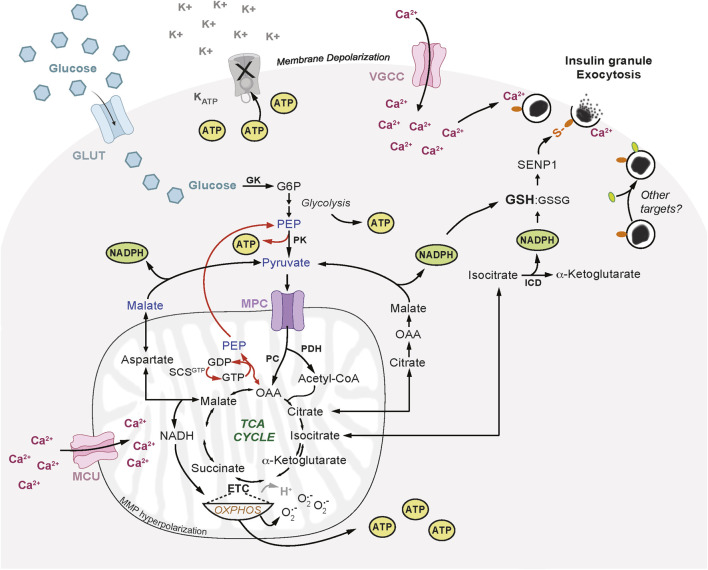
Mitochondrial Pathways Required for Insulin Secretion. Glucose enters pancreatic β-cells through glucose transporters (GLUT) and is phosphorylated by glucokinase (GK) to form glucose-6-phosphate (G6P) which is further metabolized in glycolysis. Pyruvate generated from glycolysis enters mitochondria via the mitochondrial pyruvate carrier (MPC), to participate in several metabolic cycles that are required for insulin secretion. Pyruvate is hydrolyzed by pyruvate dehydrogenase (PDH) to form acetyl-coA and contribute to the production of tricarboxylic acid (TCA) cycle intermediates. TCA intermediate reactions supply the electron transport chain (ETC) with reducing equivalents and contribute to ATP synthesis driven by the proton motive force generated by mitochondrial membrane hyperpolarization. Pyruvate can also enter the TCA cycle via pyruvate carboxylase (PC) to produce oxaloacetic acid (OAA) and regenerate pyruvate via the GTP-specific succinyl-CoA synthetase (SCS-GTP) and GTP-phosphoenolpyruvate (PEP) cycle. Mitochondrial-cytosolic isocitrate exchange can generate nicotinamide adenine dinucleotide phosphate (NADPH) via isocitrate dehydrogenase (ICD) which increases reduced glutathione (GSH) and lowers oxidized GSH (GSSG). The anapleurotic malate-aspartate and citrate-malate shuttles can support additional NAPDH synthesis. GSH can activate sentrin/SUMO-specific protease-1 (SENP1) that deSUMOylates proteins on insulin granules and enhances granule docking and exocytosis at the plasma membrane. There may be targets and modifications beyond deSUMOylation that contribute to insulin granule docking and exocytosis. Mitochondrial membrane potential (MMP) decreases or hyperpolarizes. Oxidative phosphorylation (OXPHOS) increases in coupling to ATP synthesis, proton leak (H^+^) is reduced, and superoxide (O_2_^•-^) production is increased. ATP generated from glycolysis and mitochondrial OXPHOS causes K_ATP_-channel (K_ATP_) closure and plasma membrane depolarization which leads to opening of voltage-gated calcium channels (VGCC) and calcium (Ca^2+^) influx. As Ca^2+^ increases in the cytosol this enhances mitochondrial Ca^2+^ uptake through the mitochondrial calcium uniporter (MCU) which may also be influenced by mitochondrial membrane hyperpolarization. Insulin granule exocytosis requires this influx in both cytosolic and mitochondrial Ca^2+^.

The events required for insulin secretion have been extensively studied in β-cell lines and rodent primary whole islets and have been comprehensively reviewed (Prentki et al., 2013; Campbell and Newgard, 2021; Merrins et al., 2022; Rutter et al., 2023). In both mouse and human β-cells, glucokinase (HKIV; GCK) plays the dominant rate-limiting role in insulin secretion as it initiates phosphorylation and breakdown of glucose (De Vos et al., 1995; Matschinsky and Wilson, 2019), and contributes to a biphasic pattern of insulin release (Henquin et al., 2015). Although, the kinetics and amplitude of response may differ slightly between mouse and human β-cells due to differential expression of glucose transporter isoforms (GLUT 1 and 3 in humans, GLUT2 in rodents) (De Vos et al., 1995; McCulloch et al., 2011; Berger and Zdzieblo, 2020). Upon glucose uptake and subsequent rises in ATP levels, K_ATP_-channels close and voltage-gated calcium channels (VGCC) open to allow for an influx of Ca^2+^ critical for the rapid first phase of insulin release. This is the electrical excitation-dependent phase of insulin secretion (Ashcroft and Rorsman, 2013; Rorsman and Braun, 2013). Glycolysis rates increase immediately and stabilize around 5 min after glucose stimulation and within another 10–15 min, generate notable levels of pyruvate apportioned for mitochondrial metabolism. This enhances production of suggested ‘coupling factors’ that potentiate the second phase of insulin release. These include ATP, TCA intermediates, mitochondrial GTP/phosphoenolpyruvate (Stark et al., 2009; Lewandowski et al., 2020), isocitrate/glutamine decarboxylation/SENP1 (Zhang et al., 2021), monoacyl-glycerol (Zhao et al., 2014; Attane et al., 2016), and acetyl- and malonyl-coA (Prentki et al., 2020) (Figure 1).

### 2.1 Mitochondrial metabolism

The impact of mitochondrial metabolism on insulin secretion is the most well-studied area of mitochondrial β-cell research and has been reviewed extensively (Maechler and Wollheim, 2000; Maechler et al., 2006; Jitrapakdee et al., 2010; Kaufman et al., 2015; Campbell and Newgard, 2021; Rutter et al., 2023). Glucose carbons are preferentially supplied to mitochondria as pyruvate, this results from downregulation of alternate or anaerobic routes of glycolysis synthesizing products other than pyruvate. For example, only ∼3% of glucose carbons enter the pentose phosphate pathway (PPP) (MacDonald, 1993), and minimal amounts are used for lactate production (Sekine et al., 1994; Schuit et al., 1997) due to low lactate dehydrogenase activity and expression in β-cells (Sekine et al., 1994; Pullen et al., 2010). Pyruvate enters the mitochondria through mitochondrial pyruvate carriers 1 and 2 (MPC1 and 2), and their genetic depletion or pharmacologic inhibition reduces glucose-stimulated insulin secretion (GSIS) in rodent and human islets (Patterson et al., 2014; Vigueira et al., 2014), underscoring the importance of mitochondrial pyruvate entry for the β-cell functional response to glucose. Pyruvate is then broken down to form acetyl-coA by pyruvate dehydrogenase (PDH). Pyruvate can also enter the tricarboxylic acid (TCA) cycle via a carboxylation reaction to form oxaloacetate by pyruvate carboxylase (PC). PDH and PC may have near equal rates in β-cells, however, PC activity positively correlates with insulin secretion and increasing glucose concentrations, unlike PDH (Khan et al., 1996; Schuit et al., 1997; Sugden and Holness, 2011) (Figure 1).

The relatively high activities of β-cell PC and the mitochondrial phosphoenolpyruvate (PEP) cycle further confine glucose to pyruvate metabolism in the mitochondria and are beneficial for insulin release, glucose homeostasis, and high fat diet (HFD)-induced insulin resistance (Abulizi et al., 2020). High PC activity is also linked to other metabolic intermediates including those involved in arginine metabolism, the urea cycle, and glutathione *de novo* synthesis which improve β-cell resilience against diabetes-related inflammation and oxidative stress (Fu et al., 2020; Fu et al., 2021). Furthermore, PC activity provides another means by which β-cells can impede lactate production, and interestingly, lactate accumulates in humans with PC deficiency (Habarou et al., 2015). Mitochondrial metabolism also impacts insulin secretion via the electron transport chain (ETC) and the TCA cycle, which generate ATP and other coupling factors essential for insulin release (Figure 1). Few studies have made direct biochemical links from mitochondrial metabolism to insulin granule exocytosis. One example is that mitochondrially-derived isocitrate, through its metabolism by cytosolic isocitrate dehydrogenase (ICD), enhances reductive potential that then activates sentrin/SUMO-specific protease-1 (SENP1), a deSUMOylating protein (Ferdaoussi et al., 2015). SENP1KO β-cells have reduced insulin exocytosis but no defects in upstream insulin events, thus the authors conclude this was due to a direct effect on exocytosis, and while the precise biochemical mechanism remains unknown, it presumably involves deSUMOylation of multiple granule-regulating proteins (Ferdaoussi et al., 2015). TCA activity can also support the malate–aspartate shuttle that converts cytosolic NADH to mitochondrial FADH and NADH, enhancing ATP production which leads to K_ATP_-channel closure (Eto et al., 1999). The malate-aspartate shuttle and glutamate dehydrogenase (GDH) may also directly impact insulin secretion via influencing glutamate levels (Vetterli et al., 2012; Gheni et al., 2014). Direct uptake of glutamate potentiated by incretins underlies the amplification of insulin secretion, since glutamate transporter knockdown within insulin granules reduces GSIS (Gheni et al., 2014). TCA anapleurotic activity could also support insulin secretion via a mitochondrial GTP-PEP cycle that operates independently of OXPHOS to enhance insulin release (Jesinkey et al., 2019). This pathway of GSIS involves mitochondrial GTP and PEP cycling (Jesinkey et al., 2019), and likely implicates cytosolic pyruvate kinase (PK) (Lewandowski et al., 2020), since PKm1/2 β-cell KO mice have no change in *in vivo* glucose tolerance and homeostasis (Foster et al., 2022). PK influence over GSIS is thought to involve ATP-dependent K^+^_ATP_-channel closure, but how and whether this activity depends on mitochondrial GTP cycling is not fully understood. The increase in ATP independent of OXPHOS presents a new model that stipulates a “silent phase” and avoids ADP deprivation via over-activity of OXPHOS, thus allowing for subsequent rounds of OXPHOS. How this impacts ADP within mitochondria while being driven by cytosolic PK which is presumed to be at the plasma membrane (PM) is uncertain. It would be interesting to see if mitochondrial heterogeneity between those located near the PM *versus* those further away might be involved. It will also be important to know whether this is part of the natural GSIS pathway for human β-cells.

At their most proximal steps, most of the pathways of mitochondrial metabolism impacting GSIS in rodent studies are conserved in human β-cells. Of note, human β-cells have lower rates of mitochondrial metabolism across various studies, although this may be explained by the lower relative abundance of β-cells in human islets or experimental caveats such as poorer islet culture quality compared to freshly isolated rodent islets. How these many arms of mitochondrial metabolism integrate in an epistatic fashion or act independently to regulate insulin secretion remains to be determined. Nonetheless, the importance of mitochondrial activity in general is evident in a study that depleted mitochondrial DNA with ethidium bromide which reduced glucose-stimulated ETC activity, ATP production, Ca^2+^ influx, and insulin secretion (Noda et al., 2002). It would be interesting to see how newer models of mitochondrial depletion, including the use of genetic tools, impact β-cell biology and insulin secretion (Stewart, 2021).

### 2.2 Mitochondrial oxidative phosphorylation and proton leak

Oxidative metabolism is favored by β-cells, this encompasses the oxidative arm of glycolysis and mitochondrial respiration, which is the consumption of oxygen molecules to produce metabolic intermediates of the TCA and ETC, and synthesize ATP. The energy in mitochondrial substrates, such as TCA intermediates, is transferred through the ETC by reducing NAD^+^ at Complex I (NADH dehydrogenase) and FAD^+^ at Complex II (Succinate dehydrogenase; SDH). This energy is ultimately used by Complex IV (cytochrome c oxidase) to reduce oxygen to water and remove electrons so that the carriers can be re-oxidized to continue the transport of energy through the ETC (Martinez-Reyes and Chandel, 2020). The proton gradient created by the ETC drives ATP synthesis via ATP synthase, and this coupling is known as OXPHOS. Mitochondrial respiration in β-cells increases to an exceptionally high level in response to increasing glucose concentrations, surpassing even muscle cells, despite an unusually high basal level of proton leak (Affourtit and Brand, 2006; Wikstrom et al., 2012). However, coupling to ATP synthesis increases upon glucose stimulation (Affourtit and Brand, 2006; Wikstrom et al., 2012) resulting in a relative reduction of leak compared to basal glucose levels (Figure 1).

It is important to consider caveats in studies on mitochondrial bioenergetics, electron, and proton leak including the penetrance of mitochondrial inhibitors and the challenge of uniformly delivering drugs to all cells within islets. Recent advances in approaches for measuring oxygen consumption in individual large-sized islets have revealed considerable heterogeneity between islets of the same donor or mouse (Taddeo et al., 2018). In addition, intact islets, as opposed to re-aggregated ones, demonstrate a higher capacity for respiration (Taddeo et al., 2018). Conducting bioenergetics analyses on single islets can improve our overall understanding of islet bioenergetics (Taddeo et al., 2018). However, even with these advancements current methods are unable to distinguish islet cell type-specific contributions to mitochondrial respiration.

Mitochondria can generate reactive oxygen species (ROS) in the form of superoxide primarily due to electron leak at Complex I and III (Zhao et al., 2019). This phenomenon is often associated with proton leak (Zhao et al., 2019). Acute mitochondrial reactive oxygen species (mtROS) can amplify GSIS, but prolonged elevated exposure impairs insulin release and β-cell survival (Graciano et al., 2011). In low glucose, limiting mtROS may prevent inappropriate insulin release (Mailloux et al., 2012). Conversely, high levels of matrix ROS can activate a supraphysiologic uncoupling protein 2 (UCP2)-dependent increase in proton leak, impairing GSIS (Mailloux et al., 2012). The impact of different sources of ROS on insulin secretion is highly context-dependent. Some studies observe amplification or positive correlation (Pi et al., 2007; Graciano et al., 2011; Rebelato et al., 2011; Mailloux et al., 2012; Lagundzin et al., 2019), while others report impairment or inverse correlation with GSIS (Sakai et al., 2003; Munhoz et al., 2016; Mukai et al., 2022). While signaling mtROS may be beneficial, excess ROS accumulation from any source can uncouple OXPHOS, decrease ATP synthesis, and elevate mitochondrial membrane potential (MMP), all of which are associated with reduced insulin secretion (Newsholme et al., 2019).

High proton leak could increase superoxide/mtROS if it exceeds OXPHOS coupling to ATP synthesis. One study found that in INS1 β-cells, high glucose decreased superoxide/mtROS production, which was attributed to pyruvate cycling activity from the malate-aspartate and citrate-isocitrate shuttle substrates (Plecita-Hlavata et al., 2020). However, it should be noted that this study used oxidation of mitoSOX dye to measure mtROS, which is an indirect measure, and its oxidation may depend on redox metal ions and/or an unknown peroxidation mechanism which may be reduced by glucose independently of OXPHOS (Kalyanaraman, 2020). Furthermore, the coupling of OXPHOS relative to oxygen consumption is elevated beyond the increase in proton leak compared to low glucose (Leloup et al., 2009; Taddeo et al., 2020). The enhanced coupling contributes to the production of signaling mtROS required to amplify insulin secretion (Leloup et al., 2009; Taddeo et al., 2020). At low glucose proton leak was shown to enhance insulin secretion independent of OXPHOS and required cyclophilin D activity, but this effect was not observed in high glucose-dependent proton leak (Taddeo et al., 2020). Proton leak may be less pronounced in human β-cells (Wikstrom et al., 2012), or high heterogeneity between islets within a human donor may impede large batch islet measurements (Taddeo et al., 2020). UCP2 was an early candidate for producing proton leak, however, UCP2KO β-cells have increased OXPHOS and ATP levels, with no change in proton leak (Zhang et al., 2001; Robson-Doucette et al., 2011). Thus, the source of leak during GSIS remains unknown but may involve cyclophilin D, a regulator of the mitochondrial permeability transition pore (Taddeo et al., 2020). Mitochondrial membrane potential (MMP) also hyperpolarizes in response to glucose (Figure 1), contributing to significant ATP production in human islets (Gerencser et al., 2016; Gerencser, 2018). The role of MMP is thought to be dependent on OXPHOS, and their contributions to insulin secretion are usually associated (Gerencser, 2018), however the precise connections linking these processes in β-cells, and whether they are bi-directional, are not known. Despite these unknowns, mitochondrial respiration is essential for the dynamic glucose-stimulated increase in ATP:ADP and GSIS in the INS1 β-cell line and mouse and human β-cells (De Marchi et al., 2014).

### 2.3 Mitochondrial calcium

Cytosolic Ca^2+^ is integral for insulin secretion and a growing body of evidence reveals that mitochondrial Ca^2+^ regulation, specifically by the mitochondrial calcium uniporter (MCU) and its associated proteins, mitochondrial calcium uptake 1 and 2 (MICU1 and MICU2), are also critical for maintaining insulin secretion (Alam et al., 2012; Georgiadou et al., 2020; Vishnu et al., 2021). The MCU transports Ca^2+^ into mitochondria in a membrane potential-driven manner that does not require ATP (Vais et al., 2020). Ca^2+^ influx into the mitochondrial matrix via MCU and MICU1 is required for oxidative metabolism of glucose and ATP generation in INS1 cells and rat islets (Wiederkehr et al., 2011; Alam et al., 2012; Tarasov et al., 2012). MCU and MICU1 are necessary for proper insulin release in INS1 cells, as knockdown of MCU or MICU1 decreases GSIS (Alam et al., 2012). Specific deletion of MCU in β-cells in mice led to significantly higher blood glucose levels upon glucose challenge due to impaired *in vivo* insulin release (Georgiadou et al., 2020). In line with these results, MICU2KO β-cells have decreased GSIS and ATP/ADP, absent mitochondrial membrane hyperpolarization, and diminished influx of cytosolic Ca^2+^ in response to elevated glucose (Vishnu et al., 2021).

The kinetics of mitochondrial matrix Ca^2+^ accumulation is delayed by seconds compared to the rapid increases in cytosolic Ca^2+^ in response to glucose (Waldeck-Weiermair et al., 2012). At rest, cytosolic Ca^2+^ is ∼50–70 nM which increases to ∼300–400 nM after glucose stimulation (Zhang et al., 2003; Wiederkehr et al., 2011), and there are large oscillations thought to be essential for GSIS and may involve mitochondrial and ER exchange. INS1 β-cell mitochondrial matrix Ca^2+^ increases from ∼100 to 200 nM to ∼600–800 nM, which is slightly higher compared to the increase in cytosolic Ca^2+^, and gradually increases without the oscillatory nature (Wiederkehr et al., 2011). Glucose-stimulated mitochondrial uptake of Ca^2+^ occurs at a constant rate and has been proposed to serve as a sink for cytosolic Ca^2+^ (Rutter et al., 2023). Such a sink would further potentiate the demand for ATP synthesis after initial increased ATP production, and increased activation of ETC and dehydrogenase by Ca^2+^ (Jouaville et al., 1999). Efflux of Ca^2+^ from mitochondria may be low in β-cells, which could further contribute to its accumulation upon glucose stimulation. For example, the efflux Na^+^/Ca^2+^ exchanger restricts mitochondrial Ca^2+^ accumulation, promotes cytosolic oscillations in Ca^2+^ and contributes to insulin release in MIN6 β-cells to a minor degree (Nita et al., 2012). Tarasov *et al.* proposed that slow mitochondrial Ca^2+^ accumulation transmits a constant, averaged signal from oscillations in cytosolic Ca^2+^ to allow for ATP synthesis, presumably via TCA cycle enzyme activation (Tarasov et al., 2013). Interestingly, when Ca^2+^ is depleted in human β-cells, glucose-stimulated ATP-coupled mitochondrial respiration is completely blocked (De Marchi et al., 2014). On the other hand, mitochondrial metabolism may also be required for mitochondrial Ca^2+^ influx, as revealed in studies that inhibited the ETC in INS1 cells and found lower glucose-induced ATP synthesis and mitochondrial Ca^2+^ influx (Jeyarajan et al., 2023). Thus, the impact of mitochondrial Ca^2+^ on insulin secretion via plasma membrane depolarization and/or mitochondrial bioenergetics and metabolism is complex and multidirectional (Zhang et al., 2003). Mitochondria and ER also exchange Ca^2+^ which may further exert control over insulin secretion (Alam et al., 2012; Klec et al., 2019; Jeyarajan et al., 2023), although these aspects are less well-defined and merit further testing in primary islets and *in vivo* models.

### 2.4 Mitochondrial dynamics and mitophagy

Mitochondrial dynamics, fission, fusion, and mitophagy/turnover are also important regulators of β-cell function, mass, and survival (Molina et al., 2009; Rutter et al., 2023). Dynamin-related protein 1 (DRP1), a regulator of fission, is required for the metabolic amplifying phase of insulin secretion and led to disrupted hyperfused mitochondrial networks yet did not have any effect on oxygen consumption nor Ca^2+^ influx when depleted in β-cells *in vivo* (Reinhardt et al., 2016; Hennings et al., 2018). Impaired insulin secretion in DRP1-deficient MIN6 β-cells was fully restored with pyruvate supplementation, indicating that the major defect is one of substrate generation as opposed to mitochondrial function (Kabra et al., 2022). This implies that control of insulin by mitochondrial pyruvate cycles is dependent on structural dynamics. Inducing mitochondrial fragmentation with mitofusin1 and 2 (MFN1/2) knockout in β-cells results in hyperglycemia, and reduced circulating insulin, mitochondrial length, MMP hyperpolarization, ATP synthesis, and Ca^2+^ influx in response to glucose (Georgiadou et al., 2022). In complementary studies, depletion of MFN1/2 found similar hyperglycemia, reductions in insulin secretion, and mitochondrial network fusion and respiration, however, these effects correlate more with effects on mitochondrial DNA (mtDNA) content rather than changes in fission/fusion (Sidarala et al., 2022). MFN1/2 knockout had minimal effects on β-cell mass, underscoring a stronger impact of mitochondrial dynamics on β-cell function (Georgiadou et al., 2022; Sidarala et al., 2022). On the other hand, depletion of optic atrophy 1 (OPA1), the mitochondrial dynamin-like GTPase controlling membrane fusion and cristae formation, decreases complex IV activity and function, impaired insulin release and ATP synthesis, and impaired β-cell proliferation and mass (Zhang et al., 2011). These studies reveal a complex role for mitochondrial dynamics in insulin secretion and β-cell mass.

Mitochondrial turnover, or mitophagy, is a homeostat for population control over mitochondria and is also a key determinant of β-cell health. Studying mitophagy has been challenging in β-cells, and its relevance was brought into question when loss of function studies on Parkin, a key regulator of mitophagy through ubiquitin-dependent autophagosome degradation, in β-cells revealed no overt phenotype (Corsa et al., 2019). However, depletion of the Parkin activator PTEN-induced putative kinase 1 (PINK1) impaired glucose uptake and yet increased basal insulin release with an overall improvement in glucose tolerance *in vivo,* thus suggesting that PINK1 mediates glucose-sensing coupling to insulin release (Deas et al., 2014). Most studies on mitophagy have been conducted in pathogenic and diabetic contexts and are briefly described in ***The Bad*** sections below and are reviewed elsewhere in greater detail (Lee et al., 2019; Blagov et al., 2023). Our knowledge of *bona fide* mitophagy in healthy β-cells may improve with the development of new tools to study mitophagy directly or using orthogonal indirect tools (Aoyagi et al., 2023; Levi-D'Ancona et al., 2023). In general, we know very little about the requirements for mitophagy at the subcellular and biochemical level in β-cells but there is compelling evidence for its importance in diabetes, and it is an expanding area we look forward to learning more about in future studies.

## 3 The Bad: mitochondrial dysfunction in diabetes

**FIGURE 2:**
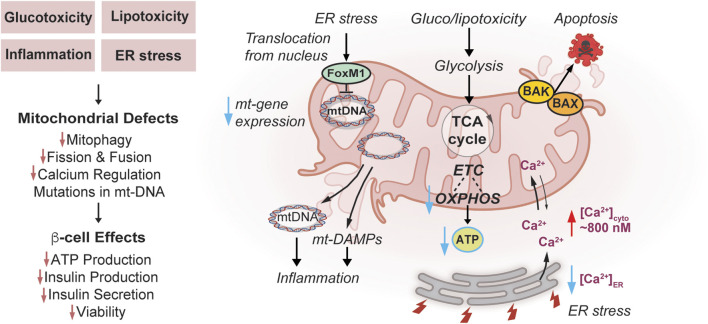
Effects of Mitochondrial Dysfunction on β-cells in Diabetes. Diabetogenic stress, such as glucotoxicity, lipotoxicity, inflammation, and endoplasmic reticulum (ER) stress impair mitochondrial turnover/mitophagy, dynamics, and calcium regulation. In addition, ER stress induced by diabetogenic stress causes FOXM1 to translocate from the nucleus to mitochondria, bind mtDNA and impair replication potentially contributing to mtDNA mutations. Inflammation can be amplified through release of mt-DNA and mt-DAMPs. Diabetogenic stresses enhance mitochondrial apoptosis pathways through Bcl-2 Associated X-protein (BAX) and Bcl-2 homologous antagonist/killer (BAK) activation. Gluco-/lipotoxic stress impairs mitochondrial ETC and OXPHOS, and lowers coupling to ATP synthesis. These stress pathways culminate in a loss of glucose-responsiveness including reduced ATP synthesis, insulin production and secretion, and diminished viability of β-cells.

Mitochondria have a powerful impact on insulin secretion and β-cell function and survival. In the context of diabetes, countless lines of evidence reveal that mitochondria are dysfunctional and may exacerbate disease progression (Figure 2). However, it is unclear if mitochondria play a direct, causal role in the development of diabetes. In the next sections, we detail the mitochondrial dysfunction in response to diabetogenic stress in terms of cellular respiration, Ca^2+^ levels, ER stress, and mitophagy.

### 3.1 Mitochondrial metabolism and bioenergetics in gluco- and lipotoxicity

Dysregulated mitochondrial bioenergetics, metabolism, and OXPHOS are frequently observed in models of T2D. A common approach is to use glucotoxic and lipotoxic culture conditions to model the chronically elevated glucose and free fatty acid (FFA) levels in T2D. This metabolic stress leads to defective insulin secretion, apoptosis, and dedifferentiation of β-cells owing to molecular causes involving a combination of mitochondrial dysfunction, autophagy impairment, inflammation, ER, and oxidative stress (Lytrivi et al., 2020). Non-diabetic human donor islets cultured in chronic hyperglycemic conditions elevated mitochondrial metabolism and ATP levels even when switched back to low glucose (Chareyron et al., 2020). This overactivity at low glucose resulted in blunted OXPHOS/ATP responses to high glucose (Figure 2), and accumulation of both PPP intermediates and glycerol-3-phosphate (Chareyron et al., 2020). Another study on human islets in culture similarly found that hyperglycemia/glucotoxicity (3 days; 25 mM glucose) initially increased the expression of mitochondrial carriers, ETC components, and mitochondrial import machinery (Jimenez-Sanchez et al., 2020). Direct effects of inhibiting mitochondrial pyruvate metabolism using 10 µM UK5099 rescued the metabolic defects and glucose induced Ca^2+^ influx, supporting a theory that enhanced early mitochondrial metabolism contributes to islet dysfunction in hyperglycemia (Chareyron et al., 2020). Unfortunately, whether these effects were MPC-dependent, and if this dose also restored insulin secretion was not evaluated, this is a relevant question because at higher doses (100 µM) it decreases insulin secretion (Patterson et al., 2014). Interestingly, lipotoxic culture conditions (3 days; 0.4 mM oleate/palmitate) have opposing effects and downregulate mitochondrial ETC gene expression (Jimenez-Sanchez et al., 2020). Lipotoxicity modeled through chronic exposure to 0.5 mM palmitate increases superoxide/mtROS production, and decreases enzymes related to carbon metabolism such as citrate synthase (Maris et al., 2013). Single-cell transcriptomic studies on rat β-cells cultured in lipotoxic conditions revealed potential upregulation in glycolysis (Aldoa, Gapdh, Pgk1, and Eno1), OXPHOS (ATP5b, Cox4i2, Cox5b, Cycs, Ndufs3, and Ndufa5), and antioxidant (Sod2 or Prdx1) pathways (Vivoli et al., 2023).

Additional gluco/lipotoxic mechanisms of β-cell death include lipid peroxidation contributing to increased iron uptake and ferroptosis (Hansen et al., 2018). Islets from patients with T2D have increased lipid peroxidation adducts (MacDonald et al., 2013) and these lipid peroxides impair mitochondrial function by damaging mtDNA, RNA, and ETC machinery (Lang et al., 2011; Ademowo et al., 2017). Pancreatic β-cells are particularly low in superoxide dismutase and catalase activity, resulting in their increased sensitivity to ROS, and rely heavily on GSH, NADPH, and thioredoxin to reduce ROS (Shalev, 2014; Miki et al., 2018; Stancill et al., 2019). Supplementation with antioxidants or TCA cycle intermediates prevented β-cell death and restored insulin secretion impaired with lipotoxic stress in INS1 cells (Boucher et al., 2004; Lee J. H. et al., 2014). However, in prolonged gluco/lipotoxic exposure (10 weeks) an elevated antioxidant response is suggested to contribute to loss of GSIS (Fu et al., 2017). These studies emphasize the importance of ROS and mitochondrial metabolism in the protective and damaging adaptive aspects of gluco/lipotoxicity-induced β-cell death.

Apoptotic cell death pathways have also been implicated, in part in gluco/lipotoxicity in β-cells. In terms of pro-apoptotic proteins both Bcl-2 Associated X-protein (BAX) and Bcl-2 homologous antagonist/killer (BAK) are expressed in β-cells and have overlapping capacities to activate cell death (White et al., 2020) (Figure 2). BAX and BAK can also amplify the unfolded protein response (UPR) to further exacerbate the effects of gluco/lipotoxicity. The pro-survival proteins BCL2 and BCL-xL are also both expressed in β-cells and when depleted, glucose-stimulated ROS increases via UCP-2-independent proton leak and enhances permeability transition pore (White et al., 2020).

The non-obese βV59M mouse, carrying an inducible Kir6.2-V59M activating mutation in the K_ATP_ channel specifically in pancreatic β-cells, also effectively models chronic hyperglycemia/hypoinsulinemia and is a tool for studying the effects of T2D-related stress. Within the islets of these mice, genes related to glycolysis, polyol, and PPP are increased, whereas inner and outer mitochondrial membrane, mitochondrial ribosomal, and ETC proteins were diminished (Haythorne et al., 2019). Metabolic studies revealed that ATP and OXPHOS were decreased alongside upregulation of PPP and accumulation of glycogen, all of which correlated with hyperglycemia in INS1 cells (Haythorne et al., 2019). While gluco/lipotoxic culture conditions clearly impair mitochondrial metabolism, function, and dynamics, the precise molecular mechanisms and causal role of mitochondrial dysfunction in diabetes in this model remains uncertain.

It is important to note that *in vitro* gluco/lipotoxicity studies have significant limitations. They are intended to model the chronic and progressive hyperglycemia and hyperlipidemia in T2D but are conducted in much shorter acute settings (years *versus* days). Additional limitations arise from concentrations used for glucose and FFAs. Higher concentrations of glucose (8–33 mM) may hinder β-oxidation and metabolism of FFAs. The concentrations of glucose used in cell culture (5–33 mM glucose) often surpass postprandial levels experienced *in vivo* in mice (8–15 mM), non-diabetic individuals (6–10 mM), and T2D humans (>10 mM glucose) (Bruce et al., 2021). FFAs are observed to range from 100 µM to >1 mM in human plasma (Huber and Kleinfeld, 2017). Ideally, *in vitro* lipotoxicity models would reflect FFA concentrations experienced by islets *in vivo*. However, measuring these concentrations is challenging due to confounding factors including release of FFA from cells, lipoprotein activity, and local delivery of FFA to islets (Lytrivi et al., 2020). Additional variability arises from technical aspects of FFAs treatment in cell culture, which include variations in the purity of mixtures and methods complexing to bovine serum albumin at differing ratios with changing affinities based on the length of carbon chains and saturation of FFAs (Lytrivi et al., 2020). In the absence of high glucose FFAs do not impair insulin production or β-cell survival (Jacqueminet et al., 2000; El-Assaad et al., 2003). In INS1 cells, lipotoxicity does not exert an effect on its own but requires high glucose (11 and 25 mM) to increase triglyceride accumulation, proton leak, and MMP (Oberhauser et al., 2020). Oleate (C18:1) has the strongest effects compared to other FFAs (palmitate; C16:0, linoleate; C18:2, and linolenate; C18:3), and all FFAs except palmitate reduce insulin secretion regardless of glucose concentration (Oberhauser et al., 2020). In this study, oleate was protective against chronic high glucose, and only high glucose increased cleaved caspase 3 (Oberhauser et al., 2020). Interestingly, β-oxidation did not increase with overloading of FFAs at any glucose concentration, suggesting that glucose oxidation is favored and presides over lipid metabolism in INS1 cells (Oberhauser et al., 2020). Human islets may recover from palmitate (2 days; 0.5 mM) or high glucose (2 days; 22.2 mM) treatment alone after a washout period returning to 5.5 mM glucose, but combined glucolipotoxicity has irreversible detrimental effects (Marselli et al., 2020). In the absence of human or clinical data, the importance of lipotoxicity, independent of glucose levels, has been challenged in the field (Weir, 2020). While FFAs have acute effects on insulin secretion, there is overall less evidence that alone they impair β-cell survival in acute *in vitro* culture settings.

### 3.2 Dysregulation of mitochondrial calcium in diabetes

As noted in Section 2.3 mitochondrial Ca^2+^ is necessary for glucose-induced insulin release. MCU depletion in mouse islets exacerbates the effects of glucolipotoxicity, an acute model of metabolic stress related to T2D, by severely diminishing mitochondrial Ca^2+^ and cytosolic ATP/ADP (Tarasov et al., 2012). MCU also helps regulate cytosolic Ca^2+^ levels and may draw Ca^2+^ into the mitochondria to alleviate Ca^2+^ overload in the cytosol which can result from lipotoxicity in T2D (Ly et al., 2020). The effects of MCU on cytosolic Ca^2+^ are likely generalized as exemplified in studies on cardiomyocytes in which MCU overexpression drastically depletes cytosolic Ca^2+^ (Drago et al., 2012). In MIN6 cells, exposure to lipotoxic stress increases expression of MCU at ambient glucose concentrations and silencing of MCU alleviates lipotoxic-induced superoxide/mtROS formation and yet accelerates cytosolic Ca^2+^ overload (Ly et al., 2020). These studies linking mitochondrial Ca^2+^ to insulin secretion indicate that the MCU complex is essential for metabolism-insulin secretion coupling, and it may protect β-cells against gluco/lipotoxic stress and improve their capacity to dissipate cytosolic Ca^2+^ overload.

### 3.3 Mitochondria and diabetogenic ER stress

Mitochondrial dysfunction in diabetes is associated with ER stress (Halban et al., 2014; Hasnain et al., 2016; Zhang et al., 2020). ER stress contributes to excessive and damaging accumulation of Ca^2+^ in the mitochondrial matrix leading to inner membrane permeabilization and apoptosis (Deniaud et al., 2008). Gluco/lipotoxicity associated with diabetes also induces ER stress and excess Ca^2+^ release into the cytosol, further impairing mitochondria and insulin secretion. Interestingly, MCU is upregulated by lipotoxic stress, which may promote detoxification of cytosolic Ca^2+^ overload whereas knockdown of MCU exacerbates Ca^2+^ overload, autophagy inhibition, and cytotoxicity in the MIN6 β-cell line (Ly et al., 2020). It was proposed that ER stress-mediated overload of cytosolic Ca^2+^ may impair autophagy and therefore mitophagy and is another route by which ER stress could impair mitochondrial function (Ly et al., 2020). ER Ca^2+^ levels are also regulated by the sarco/endoplasmic reticulum calcium ATPase (SERCA), which pumps Ca^2+^ into the ER and is dependent on ATP for activity. In healthy β-cells, ER Ca^2+^ can change from 200 to 500 µM in low (5 mM) to high (20 mM) glucose, respectively, and this increase and range of concentrations are required for proper protein folding in the ER (Tengholm et al., 1999; Xu et al., 2015; Idevall-Hagren and Tengholm, 2020). Pharmacologic ER stress increases cytosolic Ca^2+^ to ∼800 nM, at the expense of ER Ca^2+^ and can inactivate SERCA (Zhang et al., 2020) (Figure 2). Pharmacologic activation of SERCA with CDN1163 increased ER and mitochondrial Ca^2+^ content, MMP, OXPHOS, and ATP synthesis, effects that were recapitulated with overexpression of SERCA2a and b (Nguyen et al., 2023). Augmented SERCA activation protected MIN6 cells from lipotoxic-induced cytosolic and mitochondrial ROS (Nguyen et al., 2023). Furthermore, enhanced organellar Ca^2+^ levels are associated with increased peroxisome proliferator-activated receptor gamma coactivator 1α (PGC1α) and TFAM expression, suggesting an improved potential for mitochondrial biogenesis (Nguyen et al., 2023). Nature provides additional evidence of the impact of ER-Ca^2+^ on β-cell dysfunction in diabetes since mutations in the gene wolframin causes diabetes mellitus and non-autoimmune insulin/β-cell-deficiency in Wolfram syndrome Type 1 (Morikawa and Urano, 2022). Interestingly, these mutations also associate with secondary mitochondrial dysfunction although their distinct contribution to diabetes development is uncertain in this context of protein misfolding (La Morgia et al., 2020).

ER stress can impact the cells in non-Ca^2+^-dependent manners as well, including the UPR. β-cells are uniquely reliant on and sensitive to ER stress (Sharma et al., 2021) because they are the only cells that produce and process high levels of insulin prohormone (Sun et al., 2015; Yong et al., 2021). Although highly associated with both T1 and T2D, it remains uncertain whether proinsulin processing defects induce or result from disease (Sharma et al., 2021). The importance of insulin processing to β-cell function is evident from the numerous mutations in the human insulin gene leading to hormone misfolding, that cause mutant INS-gene-induced diabetes of youth (MIDY) (Haataja et al., 2021). Importantly, serine/threonine-protein kinase/endoribonuclease IRE1 (IRE1), a regulator of UPR, is required for enhancing insulin biosynthesis in the ER in response to transient glucose, however, in chronic glucotoxic conditions it becomes hyperactive and enhances ER stress (Lipson et al., 2006). Blocking the UPR by overexpressing a novel regulator of IRE1, EDEM1, improves glycemia and insulin secretion in rats treated with streptozotocin as a model of β-cell loss (Flintoaca Alexandru et al., 2023). The transcription factor FoxM1 is activated by ER stress in β-cells, whereby it translocates from the nucleus to the mitochondria, directly binds mtDNA, and impairs mitochondrial gene transcription, respiration, and ATP synthesis (Kobiita et al., 2023) (Figure 2). FoxM1 high-expressing β-cells are protected from ER and glucotoxic stress by an overall reduction in protein synthesis and mitochondrial activity and are associated with increased cell cycle progression and cell survival (Kobiita et al., 2023). Taken together, these studies reveal that ER stress can impair mitochondrial function and contribute to diabetes pathogenesis.

### 3.4 Defective mitophagy in diabetes

Diabetes, particularly T2D, can be referred to as a disease of aging, in that cumulative dysfunction over time can impair β-cells, culminating in an increased prevalence of 1 in 3 people over 65 years old developing T2D (Kalyani et al., 2017). In addition, certain aspects of aging may enhance the risk of developing T2D in elderly populations, such as sarcopenia (Mesinovic et al., 2019; Chen et al., 2023), which may be dependent on declining mitochondrial function with age. Mitophagy has the clearest connection to diseases of aging since its impacts are less pronounced acutely, and rather develop over time as damaged mitochondria accumulate. In human T2D islets, GSIS and ATP are diminished and associated with loss of mitochondrial hyperpolarization, swollen cristae, and increased mitochondrial density volume (Anello et al., 2005), and these functional and structural defects imply reduced mitophagy. In response to HFD, enlarged islets have elevated mitophagy due to hypoxia and the hypoxia-inducible factor 1-ɑ (HIF-1ɑ)/BCL2 interacting protein 3 (BNIP3) axis (Aoyagi et al., 2023). Despite the increased mitophagy, damaged mitochondria still accumulated more in enlarged islets, likely due to increased ROS. Another regulator of mitophagy via Parkin is the mitochondrial Rho protein 1 (MIRO1), since its depletion in β-cells leads to mitochondrial dysfunction, reduced insulin release, and reduced mitophagy (Chen et al., 2017). Expression of MIRO1 was reduced in islets from T2D patients and *db/db* diabetic mice, supporting a possible role of mitophagy deficiency in the pathogenicity of T2D. Transcription factor EB (TFEB) may regulate mitophagy via its established role in autophagy gene expression and lysosome formation (Oh et al., 2023). In β-cells treated with rotenone or oligomycin, TFEB translocates to mitochondria and correlates with lysosomal Ca^2+^ release and mitophagy (Park et al., 2022). TFEBKO mice were sensitized to HFD-induced glucose intolerance and impaired insulin secretion, and had reduced mitophagy, cytochrome c release, and oxygen consumption (Park et al., 2022). The role of mitochondrial transcription factor B2 (TFB2M), another regulator of mitochondrial gene expression, was observed in mouse TFB2M-KO β-cells which had impaired mitophagy via defects in autophagosome-lysosome fusion, and this was associated with mitochondrial dysfunction, and deficient insulin release (Nicholas et al., 2017).

The T1D susceptibility gene, C-type lectin domain containing 16a (Clec16a) plays a prominent role in mitophagy in β-cells through the PINK1 target Nrdp1 E3 ubiquitin ligase (Sidarala et al., 2020). Clec16a KO islets have defective mitochondria with impaired oxygen consumption and ATP levels and reduced insulin secretion (Soleimanpour et al., 2014). Pro-inflammatory cytokines induce mitophagy as a protective response, and mitophagy-deficient β-cells accumulated dysfunctional mitochondria and had increased cell death, whereas overexpression of Clec16 rescued viability and mitophagy in human β-cells (Sidarala et al., 2020). Furthermore, β-cell-specific Clec16a-KO mice developed more severe diabetes after multiple low-dose streptozotocin treatments, a model of β-cell loss in T1D (Sidarala et al., 2020).

Another potential player is the protein Nor1, a member of the Nr4a subfamily of nuclear receptors, whose expression is increased in T2D islets and correlated with increased β-cell apoptosis (Close et al., 2020). Upon pro-inflammatory cytokine treatment, Nor1 translocates to mitochondria and is associated with decreased GSIS, glucose oxidation, and ATP synthesis, possibly due to increased mitochondrial fragmentation and mitophagy (Close et al., 2020). Mitophagy driven through these pathways may be independent of Parkin since ParkinKO β-cells display no phenotype (Corsa et al., 2019), however, Parkin may yet be relevant under diabetogenic stress conditions inducing β-cell loss (Hoshino et al., 2014).

## 4 The interplay of inflammation and mitochondria in diabetes

Autoimmunity in T1D involves a complex interplay of innate and adaptive immune cell effectors contributing to β-cell inflammation at different stages of disease. Cytotoxic T-cells have the capacity to directly eliminate β-cells through mechanisms involving perforin and granzyme B, engaging mitochondrial cell death pathways (Thomas et al., 2010; Chen et al., 2018). MtROS in both β-cells and immune effector cells can also play a pivotal role in inflammation and β-cell death in T1D (Chen et al., 2018). For instance, autoreactive T-cell antigen-specific activation is dependent on mtROS which subsequently hyperpolarizes MMP and can impair mitochondrial function (Chen et al., 2018). Scavenging excess mtROS in diabetogenic T-cells reduces their shift to aerobic glycolysis and impairs their ability to induce diabetes following adoptive transfer (Previte et al., 2017). Recently, tryptophan metabolism was found to protect against *in vitro* T-cell mediated β-cell killing through PD-L1 agonism (Rachdi et al., 2023). This protection may involve mitochondrial metabolism, as exogenous tryptophan may enhance NAD and mitochondrial function via the kynurenine pathway (Castro-Portuguez and Sutphin, 2020). In addition, the mitochondrial gene NADH dehydrogenase 2 (mt-ND2), a T1D-associated SNP, demonstrates a resistant allele that prevents diabetes in NOD mice, a model of T1D, following adoptive transfer of diabetogenic T-cells, and reduces T-cell mediated killing of a β-cell line *in vitro* (Chen et al., 2011). Macrophages also contribute to β-cell inflammation in T1D, potentially by presenting β-cell antigens, releasing mtROS, and amplifying proinflammatory cytokines signals leading to β-cell destruction (Padgett et al., 2013; Burg and Tse, 2018; Chen et al., 2018; Zirpel and Roep, 2021).

In T2D, autoimmunity-like inflammation termed ‘sterile inflammation’ has recently emerged as an etiology. This refers to inflammation occurring in the absence of overt pathogen or infection. Mitochondrial dysfunction, mtDNA mutations, and ROS have been implicated in the process of ‘inflammaging’ a state of chronic inflammation associated with aging and involved in T2D development (Zuo et al., 2019; Dabravolski et al., 2021). Another term used to describe chronic inflammation in T2D is ‘metaflammation’, a state of inflammation stimulated by an excess of nutrients and aging (Prattichizzo et al., 2018). Factors and cytokines derived from adipose in obesity, such as TNF and IL-6, may amplify β-cell sterile inflammation and NFkB-signaling contributing to T2D development (Biondi et al., 2022). On the other hand, cellular senescence, and senescence-associated secretory phenotype (SASP) cytokines (IL-1ɑ/β, IL-6, and TNF) are chronically elevated in diabetes patients, suggesting that senescence also influences inflammation associated with T2D and excess nutrients (Prattichizzo et al., 2016). Islet-resident macrophages are the primary immune cell effector implicated in T2D-related β-cell inflammation (Ying et al., 2019). We anticipate that macrophages may respond to metabolic signals originating from β-cells, including mitochondrially-derived metabolites and factors. Efferocytosis of dying β-cells in mouse models of β-cell loss stimulates macrophages to secrete insulin growth factor 1 (IGF-1) potentially enhancing β-cell renewal and playing a role in β-cell mass expansion in prediabetes and early phases of T2D (Nackiewicz et al., 2020). Islet-resident macrophages increase mitochondrial OXPHOS gene expression following β-cell loss in mice (Nackiewicz et al., 2020).

Mitochondria can directly stimulate inflammation through the release of damage-associated molecular patterns (mtDAMPs) (Wang et al., 2023). Cytosolic free mtDNA can act as a potential DAMP (Figure 2), as it is particularly sensitive to damage by ROS because it lacks histones for mtDNA protection and has no apparent mtDNA repair response (Yakes and Van Houten, 1997). Increasing studies have documented that release of mtDNA activates the cyclic GMP-AMP synthase - stimulator of interferon genes (cGAS-STING) pathway triggering adipose inflammation in obesity and T2D (Bai et al., 2017; Bai and Liu, 2019; Bai et al., 2020). In mice fed HFD, mt-DNA release induced β-cell senescence and was dependent on cGAS-STING activation (Yakes and Van Houten, 1997). However, STING may play additional roles in β-cell GSIS, as STING knockout mice, although protected from insulin resistance exhibited diminished insulin responses (Qiao et al., 2022). Mitochondrially-encoded peptides are another means by which mitochondria may influence inflammation. The mitochondrial peptide MOTS-c (mitochondrial open reading frame of the 12S rRNA type-c) is suggested to have anti-inflammatory properties, protecting NOD mice against T-cell-mediated β-cell destruction through a mechanism involving T cell receptor (TCR) and mammalian target of rapamycin (mTOR) signaling (Kong et al., 2021). Circulating levels of MOTS-c may also be reduced in humans with T2D in correlation with poor glycemic control (Du et al., 2018; Kong et al., 2023).

Mitochondria can also play a role in molecular signaling of inflammation-induced β-cell death although a precise singular molecular mechanism remains elusive. Pro-inflammatory cytokines are frequently used to model β-cell death due to inflammation in T1 and T2D. Blocking apoptosome formation with knockdown of apoptotic protease activating factor 1 (APAF-1) does not protect INS1 cells from cytokine-mediated cell death (Collier et al., 2011). However, depletion of mt-DNA and thus mitochondria, from a human β-cell line completely abolishes cytokine-induced cell death (Lightfoot et al., 2011). In another human β-cell line, cytokines were similarly found to induce caspase-mediated apoptosis (Frorup et al., 2023). Inflammation-induced β-cell death may involve species-specific mechanisms. Currently, the precise mechanism is not fully defined, and the *in vivo* relevance is undefined (Usmani-Brown et al., 2019). Pro-inflammatory cytokines stimulate inflammatory signaling via mitochondria, impair OXPHOS, induce mitophagy and increase mtROS (Lambelet et al., 2018; Sidarala et al., 2020). Human islets treated with pro-inflammatory cytokines (IL1β, TNF, IFNγ) have altered mitochondrial metabolite pathways (reduced pyruvate carboxylation and arginine; increased citrulline and kynurenine), diminished mitochondrial protein turnover, and increased mitochondrial oxidation of glutathione (Garcia-Contreras et al., 2017; Fu et al., 2020; Fu et al., 2021). In INS1 cells, proinflammatory cytokines restrict GSIS by impairing pyruvate oxidation, but this occurs independently of coupling to ATP synthesis (Barlow et al., 2018). Collectively, the evidence described in this section indicates that mitochondria influence intrinsic β-cell demise and immune effector activity thereby influencing β-cell inflammation in both T1 and T2D. Our understanding is still preliminary and more detailed molecular analyses of the role of mitochondria in diabetic inflammation are needed in future studies.

## 5 Other pathways influencing mitochondria in β-cells

Few mitochondrial proteins, other than metabolic enzymes/respiratory complex components, have been tested in loss of function studies in pancreatic β-cells. Table 1 summarizes the mitochondrial and non-mitochondrial genes/proteins described in this section and their roles in β-cells. Deficiency in the expression of the mitochondrial inner membrane protein, MPV17, has been linked to ROS accumulation, mtDNA mutations, and cell death in different cell types. In contrast, β-cell-specific MPV17 knockout mice are protected from apoptosis induced by diabetogenic paradigms (STZ, INS2Akita mutation) associated with T1D, thus it may have a proapoptotic role in β-cells (Tang et al., 2023). Several mitoribosomal proteins (MRPs) are downregulated in T2D islets, and depletion of MRPL59 in haploinsufficient mice led to insulin secretion defects and exacerbated hyperglycemia in response to HFD (Hong et al., 2022). The transcription factor B1 mitochondrial (TFB1M) is linked to the development of T2D by reducing insulin secretion and promoting hyperglycemia (Koeck et al., 2011). Knockdown of a nuclear TFB1M homolog, dimethyladenosine transferase 1 homolog (DIMT1), a ribosomal RNA methyltransferase, reduces levels of OXPHOS, ATP synthase, ETC genes, and also disrupts mitochondrial function, MMP hyperpolarization, and insulin secretion in response to glucose (Verma et al., 2022).

**TABLE 1:** Other mitochondrial and non-mitochondrial proteins impacting β-cell mitochondrial function. Protein name and localization verified on Uniprot, general physiologic roles and β-cell-specific roles are described.

Protein	Location	General physiologic role	β-cell-specific roles
MPV17 (MPV17L2)	Mitochondrial inner membrane	ROS balance; maintains, ETC, MMP, and folate metabolism Madungwe et al. (2020), Jacinto et al. (2021), Spinazzola et al. (2006), Alonzo et al. (2018)	• KO mice reduces apoptosis in diabetic stress Tang et al. (2023)
MRPL59 (CRIF1, PRG6, PLINP1)	Mitochondrial inner membrane	Insertion of nascent proteins into inner mitochondrial membrane for, ETC complex; positive and negative regulation of proliferation Kim et al. (2012), Horikoshi et al. (1999), Nakayama et al. (2007)	• Decreased expression in human T2D islets; haploinsufficient mice have decreased insulin secretion and increased hyperglycemia Hong et al. (2022)
TFB1M (mtTFB1)	Mitochondria	Mitochondrial transcriptional co-activator of TFAM; tissue-specific methylation of mitochondrial rRNA (16S and 12S) McCulloch et al. (2002), Seidel-Rogol et al. (2003), Metodiev et al. (2009), Lee et al. (2015b)	• SNP in gene increases risk of T2D in humans; expression correlates with GSIS; Haploinsufficient mice have decreased GSIS Koeck et al. (2011)
DIMT1	Nucleoli, cytoplasm	Methylation of 18s rRNA, ribosome assembly Zorbas et al., 2015	• Knockdown in human cell lines and rat islets reduces OXPHOS, ATP synthesis, and GSIS Verma et al. (2022)
PAK1 (Alpha-PAK)	Cytoplasm, Plasma membrane, Nucleus	Integrin and receptor-type kinases signaling; cell structure, adhesion, migration, proliferation, apoptosis, mitosis, and vesicle transport Harms et al. (2018), Manabe et al. (2002), Rider et al. (2007), Zhou et al. (2003), Zhou and Kramer (2005)	• Reduced in islets of T2D patients Wang et al. (2011)
			• Overexpression in human islets reduces ER stress markers; KO in mice reduces glucose clearance, GSIS, and complex I; increases apoptosis and ROS Ahn et al. (2021)
			• MIN6 cell proliferation Chen et al. (2013)
CD63 (LAMP-3)	Cell Surface	Surface receptor for TIMP1, integrin signaling and activation of ITGB1 Lee et al. (2014b), Tugues et al. (2013)	• Decreased expression in HFD mice and T2D; increased GSIS, OCR in high expressing mouse and human β-cells Rubio-Navarro et al. (2023)
			• Insulin degranulation; KO mice had increased GSIS Pasquier et al. (2019)
AMPK (PRKAA1/2)	Cytoplasm, Nucleus	Energy sensor, protein kinase, metabolism, autophagy, cell growth, mitochondrial homeostasis Herzig and Shaw (2018), Towler and Hardie (2007), Hardie (2007)	• β-cell KO have reduced insulin content, mild hyperglycemia and increased proliferation Sun et al. (2010)
			• AMPK activation protects against ER and mitochondrial defects, and β-cell apoptosis in diabetic stress Wikstrom et al. (2013)
LKB1 (STK11, PJS)	Cytoplasm, Nucleus	Energy sensor, protein kinase, activates AMPK family members, cell metabolism, cell polarity, apoptosis, and DNA damage response Karuman et al. (2001), Gurumurthy et al. (2010)	• β-cell KO in mice increases insulin secretion, β-cell size, and mass, causes mitochondrial defects Swisa et al. (2015), Granot et al. (2009), Fu et al. (2015), Fu et al. (2009)
MOTS-c (MT-RNR1)	Mitochondria, Plasma membrane, Nucleus	Metabolic homeostasis, protects against obesity and insulin resistance, mutation associated with T2D Lee et al. (2015a), Zempo et al. (2021)	• Anti-inflammatory properties in NOD mice, reduces T-cell-mediated β-cell death Kong et al. (2021)
			• Reduced in T2D associated with poor glycemic control Du et al. (2018)
BAX (BCL2L4)	Cytoplasm, Nucleus, Mitochondrial outer membrane	Pro-apoptotic Pena-Blanco and Garcia-Saez (2018)	• Activates mitochondrial apoptosis White et al. (2020), McKenzie et al. (2010)
			• Amplifies UPR with BAK to exacerbate gluco/lipotoxicity White et al. (2020)
BAK (BAK1, BCL2L7, CDN1)	Mitochondrial outer membrane	Pro-apoptotic Chittenden et al. (1995)	• Activates mitochondrial apoptosis White et al. (2020)
			• Amplifies UPR with BAX to exacerbate gluco/lipotoxicity White et al. (2020)

The serine/threonine p21-activated kinase 1, PAK1, is required for β-cell replication mass and is reduced in T2D islets (Wang et al., 2011; Chen et al., 2013). β-cell-specific depletion of PAK1 impaired glucose tolerance and elevated β-cell apoptosis in mice. PAK1KO β-cells have fewer mitochondria, reduced GSIS, reduced mitochondrial ETC (complex I), and elevated ROS, whereas overexpression of PAK1 in human T2D islets reduces markers of ER stress (Ahn et al., 2021).

Single-cell transcriptomics studies on islets revealed a β-cell subset expressing high levels of CD63 and mitochondrial genes (Rubio-Navarro et al., 2023). This population was diminished in islets from HFD mice, *db/db* mice, and T2D human islets (Rubio-Navarro et al., 2023). Pseudo-islets generated from FACS-purified CD63 high cells had increased GSIS, enhanced mitochondrial activity, and restored glycemia when transplanted in NOD SCID diabetic mice compared to CD63 low-expressing pseudo-islets (Rubio-Navarro et al., 2023). On the other hand, lysosomal CD63 may contribute to insulin degranulation in *ob/ob* diabetic mice (Pasquier et al., 2019). In INS1 cells, *in vitro* stress-induced degranulation required CD63, and islets from CD63 knockout mice had reduced degranulation and increased GSIS (Pasquier et al., 2019). It would be helpful to know in future studies if CD63 localization determines differential effects on β-cell health, it is possible that CD63 plays a role outside of insulin granules and/or lysosomes producing beneficial effects.

Adenosine monophosphate–activated protein kinase (AMPK) is a master regulator of cellular energy sensing and as such plays homeostatic roles in regulating mitochondrial structure and function (Hardie et al., 2016; Herzig and Shaw, 2018). In β-cells, AMPK attenuates insulin release by regulating β-cell mass and insulin content via its ability to inhibit mTOR and yet has limited effects on acute insulin secretion during high glucose when AMPK is inactivated (Fu et al., 2013; Rourke et al., 2018). Double knockout of AMPK alpha 1 and 2 catalytic subunits revealed that AMPK depletion leads to mild hyperglycemia and insulin deficiency, increased replication but reduced β-cell size (Sun et al., 2010). Loss of energy sensing LKB1, an upstream AMPK kinase, increases insulin secretion, β-cell size and mass, and yet also leads to profound mitochondrial defects (Fu et al., 2009; Granot et al., 2009; Fu et al., 2015; Swisa et al., 2015). The defects in mitochondrial metabolism and respiration are dependent on a block in pyruvate metabolism, as respiration was restored with pyruvate supplementation (Fu et al., 2015). It is unlikely that the mitochondrial effects of LKB1 are due to AMPK given the lack of overlapping phenotypes between their respective knockout models. Interestingly, AMPK activation can prevent defects in ER and mitochondrial morphology and β-cell apoptosis during lipotoxic stress in a manner dependent on DRP1 (Wikstrom et al., 2013). Postnatal developmental studies found that β-cell maturation during weaning relies on switching from mTOR to AMPK signaling that promotes a shift to oxidative metabolism and enhanced mitochondrial biogenesis as β-cells mature (Jaafar et al., 2019). In *db/db* T2D-like diabetic mouse islets, the mTOR to AMPK switch is reversed and associated with a loss of oxidative metabolism and de-differentiation of β-cells (Jaafar et al., 2019). In addition, chronic hyperglycemia enhances mTOR and inhibits AMPK which is associated with decreased PDH activity and may underlie reduced oxidative metabolism during T2D-related diabetogenic metabolic stress (Haythorne et al., 2022). The role of AMPK, LKB1, and mTOR in regulating β-cell mitochondria is still under development with unanswered questions on the contribution of potential direct mitochondrial targets or cellular energy sensing resulting in these effects.

## 6 Monogenic forms of mitochondrial diabetes

Additional evidence of the impact of mitochondria on human β-cell function is evident from mutations or depletions in mtDNA that cause mitochondrial diabetes. This form of diabetes can also result from mutations of genes encoding mitochondrial proteins in the nuclear DNA. It is estimated that over 80% of pathogenic mutations in mtDNA occur in the MT-TL1 gene, wherein the m.3243A>G mutation is associated with maternally inherited diabetes and deafness (MIDD) and mitochondrial encephalopathy with lactic acidosis and stroke-like episodes (MELAS) (Ryytty and Hamalainen, 2023). Patients with MIDD have a higher risk of developing diabetes, and the m.3243A>G mutation is a biomarker that follows a maternal inheritance pattern (Zhang et al., 2015). Another pathogenic mutation can occur in MRPs, specifically, patients with mutations in MRPL9, MRPL27, and MRPL45 have an increased risk of developing diabetes (Sylvester et al., 2004; Hong et al., 2022). In general, in these diseases, symptoms manifest as mutations in mtDNA accumulate in tissues with a high reliance on oxidative metabolism, such as brain/central nervous system, muscle, pancreas and kidneys. Diabetes is the most common endocrinopathy, however clinical symptoms vary widely, likely due to heteroplasmic mutations with the presence of wild-type and mutated mtDNA (Russell et al., 2020). The heterogeneity makes it difficult to isolate precise disease mechanisms, and even the same mutations have high clinical heterogeneity. Disease onset is thought to result when a critical threshold of mtDNA mutation is reached on a per-cell basis (Russell et al., 2020). Experimental therapeutic strategies are exploring the reversal of mitochondrial defects (increasing OXPHOS, mitophagy, mitochondrial biogenesis, and antioxidants) rather than treating the clinical symptoms. Mitochondrial diabetes development is primarily due to β-cell deficiencies and unlike T2D, it less often involves insulin resistance and is frequently treated with insulin supplementation (Karaa and Goldstein, 2015).

## 7 Mitochondria in stem cell-derived β-cells/islets

The low availability of human islets is a major roadblock for islet cell transplantation therapy, which is the only cellular strategy to replenish insulin supply in T1D patients. The development of stem cell (SC) -derived pancreatic islets and β-cells provides a promising solution for therapeutic challenges (Pagliuca et al., 2014; Rezania et al., 2014). Additional benefits of this system include the generation of a universal donor, the potential for genetically modifying these cells to evade attack by the immune system, improved functional capacity, and a safety switch that can prevent aberrant replication or outcomes. SC-islets generated *in vitro* remain as immature β-cells and only fully mature *in vivo* after transplantation (Pagliuca et al., 2014; Rezania et al., 2014). SC-islet maturity is evaluated *in vitro* by assessing GSIS, which is significantly reduced and lacking in biphasic pattern compared to primary islets. Studies on SC-islet responses to glucose revealed that SC-islets have a significantly lower capacity for OXPHOS in response to glucose, likely due to a block in glycolysis that reduces pyruvate synthesis and impairs mitochondrial metabolism and supplementation with mitochondrial substrates pyruvate and succinate improves *in vitro* GSIS (Davis et al., 2020). Another study found metabolic deficits were notable in glucose-derived TCA intermediates and glutathione in SC-islets compared to primary islets (Balboa et al., 2022). Cell clustering *in vitro* seems to improve oxygen consumption, mitochondrial biogenesis, and insulin secretion, implying enhanced SC-derived β-cell maturation (Nair et al., 2019). Whether enhancing mitochondrial metabolism and OXPHOS can actively improve β-cell differentiation in SC-derived islets or if reduced mitochondrial function is simply a consequence of immature β-cells is an interesting open question.

## 8 The ugly: are mitochondria a cause or effect of diabetes?

It is exceptionally challenging to separate mitochondrial defects that are consequential *versus* causative in diabetes. Numerous observations of mitochondrial defects co-segregate with pancreatic β-cell dysfunction in diabetes, but few have tested whether improving the mitochondrial defects alone can ameliorate or reverse diabetes. We emphasize that the following aspects of mitochondria are supported by strong evidence in playing a direct, causal role in β-cell health and dysfunction in diabetes as opposed to merely being a consequence of it: Ca^2+^ uptake (Alam et al., 2012; Tarasov et al., 2012; Vishnu et al., 2021), bioenergetics (Zhang et al., 2011; Inoue et al., 2022; Lang et al., 2023), metabolism (Patterson et al., 2014; Vigueira et al., 2014; Fu et al., 2020; Lewandowski et al., 2020; Fu et al., 2021), mitophagy (Soleimanpour et al., 2014; Chen et al., 2017; Sidarala et al., 2020), and fission/fusion (Twig et al., 2008; Molina et al., 2009; Reinhardt et al., 2016; Hennings et al., 2018; Georgiadou et al., 2022; Sidarala et al., 2022) and mitoribosomes/translation (Hong et al., 2022; Verma et al., 2022). In gluco/lipotoxic stress, β-cell death is preventable with antioxidants and cellular damage is dependent on ROS, which is mainly generated in mitochondria, thus mitochondria can be attributed a causal role in this *in vitro* model of metabolic stress.

Cause *versus* effect is difficult to assess in mitochondrial bioenergetics and metabolism due to the numerous intermediate steps and bidirectional pathways. One of the key biochemistry tools that is lacking in the β-cell field is the ability to test predictions on purified mitochondria. The challenges include low starting material when isolating β-cells out of whole islets, high potential for insulin granules to contaminate mitochondrial preps, and of contamination from other islet cell types. In addition, an integrated understanding of the role of mitochondria in GSIS would benefit from more electrophysiologists working together with mitochondrial metabolism and bioenergetics experts (Nicholls, 2016). There is more evidence for the role of mitochondria in T2D, however, in the context of T1D, the heavy importance of β-cell loss implicates mitochondrial cell death pathways, for more on these aspects we refer the reader to other reviews (Johnson and Luciani, 2010; Vig et al., 2021). It is our opinion that the evidence generally supports that mitochondrial cell death contributes to mechanisms of β-cell death in diabetes.

## 9 Conclusion and additional considerations

In conclusion, the intricate interplay between mitochondria and diabetes, particularly in the context of pancreatic β-cells, underscores the pivotal role these organelles play in maintaining cellular homeostasis and function. Mitochondrial dysfunction is synonymous with β-cell dysfunction in diabetes, it impairs insulin secretion and sensitizes β-cells to an environment high in metabolic/nutrient stress and cell death. Understanding the molecular mechanisms governing mitochondrial function in pancreatic β-cells is essential for developing targeted therapeutic interventions aimed at preserving or restoring their vital role in glucose homeostasis. One promising candidate is Metformin, which improved mitochondrial function/fitness in peripheral blood mononuclear cells and reduced hyperglycemia in naïve T2D patients treated for 3 months (Bhansali et al., 2020). Observations in this study suggested that Metformin increased mitophagy and reduced mtROS and MMP (Bhansali et al., 2020).

Improvements in technology and the development of new tools to study mitochondria should improve our understanding of their causal roles in β-cell health and demise. An example is advances in Cryo-EM-Tomography allow us to visualize the entire β-cell mitochondrial network and it would be interesting to see how/if it changes in diabetes and in response to diabetogenic stress (hyperglycemia, inflammation, ER, oxidative). It would also be interesting to test how the mitochondrial network structurally impacts insulin granule arrangement, as well as lipid droplet dynamics, a feature more prominent in human β-cells. Mitochondrial heterogeneity is another important consideration relevant to β-cell health, metabolism, function, and survival, as we did not describe this area, we refer the reader to a recent review (Ngo et al., 2021). Much less is known about the role of mitochondria in other islet cell types, however, these are likely to be important as other islet cells are responsive to glucose, have alternate hormonal action to β-cells, and can impact β-cells activity through a paracrine- and glucose-dependent manner (Capozzi et al., 2019). Another area of interest is the study of inter-organ crosstalk (Evans and Wei, 2022; Langlois et al., 2022), for example, understanding how the gut microbiome impacts β-cell function, survival and inflammation and the role of mitochondria and related-secreted factors/exosomes is an understudied area (Fernandez-Millan and Guillen, 2022; Wei et al., 2023). Overall, our current understanding supports that mitochondrial bioenergetics, metabolism, structure, and biomass are determinants of β-cell health and their dysfunction likely plays a causal role in diabetes development. Future studies using new technologies to study mitochondria in primary β-cells will greatly enhance our knowledge of the importance of these dynamic organelles in β-cell health and diabetes.

## Figures and Tables

**Table:** 

**ADP**	adenosine diphosphate
**AMPK**	adenosine monophosphate protein kinase
**APAF-1**	apoptotic protease activating factor 1
**ATP**	adenosine triphosphate
**BAX**	Bcl2-associated X protein
**BNIP3**	Bcl-2-interacting protein 3
**Ca2+**	calcium
**Clec16a**	C-type lectin domain containing 16a
**cGAS-STING**	cyclic GMP-AMP synthase - stimulator of interferon genes
**DIMT1**	dimethyladenosine transferase 1
**DRP-1**	dynamin-related protein 1
**ER**	endoplasmic reticulum
**ETC**	electron transport chain
**FFA**	free fatty acids
**GCK/HKIV**	glucokinase/hexokinase IV
**GDH**	glutamate dehydrogenase
**GLUT1/2/3**	glucose transporters 1/2/3
**GSIS**	glucose-stimulated insulin secretion
**GTP**	guanosine triphosphate
**HFD**	high fat diet
**HIF-1α**	hypoxia-inducible factor 1-α
**ICD**	isocitrate dehydrogenase
**IFNγ**	interferon gamma
**IGF-1**	insulin growth factor 1
**IL-1⍺/β**	interleukin 1 alpha/beta
**IL6**	interleukin 6
**MCU**	mitochondrial calcium uniporter
**MELAS**	mitochondrial encephalopathy associated with lactic acidosis and stroke-like episodes
**MFN1/2**	mitofusin 1/2
**MICU1/2**	mitochondrial calcium uptake 1/2
**MIDD**	maternally inherited diabetes and deafness
**MIRO1**	mitochondrial Rho protein 1
**MMP**	mitochondrial membrane potential
**MPC1/2**	mitochondrial pyruvate carriers 1/2
**MOTS-c**	mitochondrial open reading frame of the 12S rRNA type-c
**MRPs**	mitoribosomal proteins
**mtDAMPs**	mitochondrial damage-associated molecular patterns
**mt-ND2**	mitochondrial gene NADH dehydrogenase 2
**mTOR**	mammalian target of rapamycin
**mtROS**	mitochondrial reactive oxygen species
**NAD/H**	nicotinamide dinucleotide/reduced
**NFκB**	nuclear factor kappa-light-chain-enhancer of activated B cells
**NOD SCID**	nonobese diabetic severe combined immunodeficiency
**OPA-1**	optic atrophy 1
**OXPHOS**	oxidative phosphorylation
**PAK1**	p21 activated kinase 1
**PC**	pyruvate carboxylase
**PDH**	pyruvate dehydrogenase
**PD-L1**	programmed cell death ligand 1
**PEP**	phosphoenolpyruvate
**PINK1**	PTEN-induced putative kinase 1
**PK**	pyruvate kinase
**PM**	plasma membrane
**PPP**	pentose phosphate pathway
**ROS**	reactive oxygen species
**SASP**	senescence-associated secretory phenotype
**SC**	stem cell
**SDH**	succinate dehydrogenase
**SENP1**	sentrin/SUMO-specific protease 1
**SERCA**	sarco/endoplasmic reticulum calcium ATPase
**T1D**	type 1 diabetes
**T2D**	type 2 diabetes
**TCA**	tricarboxylic acid cycle/citric acid cycle
**TCR**	T cell receptor
**TFB1M**	mitochondrial transcription factor B1
**TFB2M**	mitochondrial transcription factor B2
**TFEB**	transcription factor EB
**TNF**	tumor necrosis factor
**UCP2**	uncoupling protein 2
**UPR**	unfolded protein response
**VGCC**	voltage gated calcium channel
